# Eulogy

**DOI:** 10.1017/S1478951525100850

**Published:** 2025-10-01

**Authors:** William Breitbart

**Affiliations:** Department of Psychiatry and Behavioral Sciences, Memorial Sloan-Kettering Cancer Center, New York, NY, USA

He fell in love with me at first sight

It was my birth day

He held me in his arms

And he recognized his past in my face

His future in my eyes.

I fell in love with him a few months later

when I discovered who he was.

My Father.

I loved him beyond measure

And he loved me unconditionally

My father took care of his children and his family.

He took care of me at moments of my greatest needs

With a loving heart and the steady commitment of fatherly responsibility

And these past years it was my turn to care for him

With a loving heart and with the steady commitment of a daughter’s responsibility

As he lay dying in his bed

I recognized my past in his face

I saw my future in his eyes

And now I bury my father

But the love continues

Beyond his life

Beyond his death

A love and caring that is eternal

